# Accuracy of Outsourced Radiology Reports in Emergency Surgical Care: Do They Provide a High-Quality, Cost-Effective Service?

**DOI:** 10.7759/cureus.73152

**Published:** 2024-11-06

**Authors:** Emmanuel Obayi, Mohammed Barghash, Ye H Aung, Zoe Furber, Shua Haq, Katie McComb, Moustafa Mansour

**Affiliations:** 1 Department of General Surgery, North Manchester General Hospital, Manchester, GBR

**Keywords:** ct (computed tomography) imaging, free-text report, outsourced radiology, radiology reporting, structured report

## Abstract

Background

Computed tomography (CT) scans play a crucial role in emergency surgical care, serving both diagnostic and prognostic functions. They significantly contribute to timely and effective patient care. This study aims to compare abdominal CT scan reports prepared by locally employed radiologists with those from outsourced radiology reporting services for patients presenting with acute surgical conditions. Additionally, the study evaluates how different reporting styles, such as free-text versus structured formats, influence the overall quality of the reports.

Methods

The study was conducted in the general surgery unit of North Manchester General Hospital, Manchester, a district general hospital in North West England. Patients admitted through the accident and emergency (A&E) under the general surgery team who underwent abdominal CT scans between August 2023 and October 2023 were included. Trauma patients and those who received non-abdominal CT scans were excluded. Local radiology reports were compared to outsourced reports using statistical analysis. The clarity and comprehensiveness of the reports were carefully evaluated, and the impact of free-text versus structured reporting formats was also considered.

Results

A total of 192 patients were included in the study, with a median age of 59 years. Compared to locally reported scans, outsourced radiology reports frequently omitted commentary on key anatomical structures, including the biliary system, spleen, kidneys, lymph nodes, mesentery and peritoneum, vasculature, bones and soft tissues, and lung bases. Reports using a structured format provided more thorough commentary on the spleen, adrenal glands, mesentery and peritoneum, and bones and soft tissues compared to free-text reports.

Conclusion

Outsourced radiology reports significantly underreported important abdominal anatomical structures. Free-text reporting was associated with significant omissions in abdominal anatomical structures, which could negatively affect clinical decision-making and management plans. Further studies are recommended to evaluate the comprehensiveness of outsourced radiology reporting and to ensure standardization in reporting, ultimately providing patients with high-quality emergency care.

## Introduction

Computed tomography (CT) scans play a crucial role in emergency surgical care. They are used in the investigation of acute abdominal pain, alongside physical examination and laboratory tests, to determine the most likely diagnosis [1]. Computed tomography scans visualise most abdominal and pelvic structures, enabling the detection of acute surgical pathologies [1]. They also contribute to prompt decision-making and appropriate management, which improves patient outcomes in acute surgical pathologies [2].

With the introduction of teleradiology in the mid-1990s, the interpretation of diagnostic scan images at geographically distant sites became possible [3]. Technological advancements and the power of market forces have driven the prevalence and availability of such practices [3]. Consequently, radiology reports are not necessarily written by locally employed radiologists. The images can be digitally transferred and interpreted by radiologists working remotely [3].

In 2023, a census published by the Royal College of Radiologists (RCR) reported that over 83% of radiology departments in the United Kingdom (UK) were outsourcing radiology reports to external companies [4]. This is due to a considerable discrepancy between service demand and the availability of the local workforce. In the same year, the combined cost of outsourcing, insourcing, and ad hoc locum staffing to meet the NHS's high demand was reported to be £276 million [4].

Reports by locally employed radiologists offer the opportunity for direct communication between clinical and reporting teams. Additionally, locally employed radiologists have direct access to patients' records within the same institution, a resource not available to outsourced reporting services [5]. Consequently, concerns about the quality of outsourced radiology reports have been frequently raised [5].

With the growing availability of electronic patient records, patients have increased access to their radiology reports [6]. Therefore, a standardised writing style reflecting the needs of both patients and referring clinicians as the primary end-readers should be considered. Two commonly used reporting styles are structured and free-text formats. Structured reports are typically organised based on anatomical organs and body systems, often written as a list, while free-text reports document findings in continuous prose [7]. Structured reports enable easy extraction and assimilation of information, whilst free-text reports are often more concise [7]. Further studies are needed to identify the optimal format for radiology reports [7].

This study aims to compare abdominal CT scan reports composed by locally employed radiologists to those composed by an outsourced radiology reporting service for patients admitted with acute surgical pathologies. This study also assesses the impact of reporting style (free-text versus structured format) on the comprehensiveness of abdominal CT scan reports.

This article was previously presented as a meeting abstract at the 27^th^ Association of Upper Gastrointestinal Surgery of Great Britain and Ireland (AUGIS) Annual Scientific Meeting on September 12, 2024.

## Materials and methods

Study design and patient selection

The study was conducted as a retrospective cohort study. The Strengthening the Reporting of Cohort Studies in Surgery (STROCSS) guidelines for observational research were followed [8]. A predefined protocol, in accordance with local Clinical Governance Unit policies, was implemented. As the study was retrospective, contained non-identifiable data, and did not meet the NHS Health Research Authority criteria for ethical review, ethical approval and patient consent were not required [9]. The study was undertaken in the general surgery unit of North Manchester General Hospital, Manchester, a district general hospital in North West England. Data were collected from the hospital’s electronic healthcare records between August 2023 and October 2023. All patients aged 18 years or older who underwent emergency abdominal CT scans were included. Patients who underwent abdominal CT for trauma, non-emergency reasons, or other imaging modalities such as ultrasound, magnetic resonance imaging (MRI), or non-abdominal CT scans were excluded.

Comparisons and outcomes

The primary outcome compared abdominal CT reports from locally employed radiologists to outsourced reports for patients with acute abdominal pathologies. We analysed and compared the comments on anatomical organs and structures, documented within the reports, between the two groups. The organs assessed included the liver, biliary system, spleen, pancreas, adrenal glands, kidneys, gastrointestinal tract, lymph nodes, vasculature, mesentery, peritoneum, bones, soft tissues, and lung bases. The study also evaluated the impact of free-text and structured reporting formats on report comprehensiveness.

Data collection

An electronic data collection proforma was developed to gather the necessary data for the study. Patient demographics, including age and gender, were recorded. The indication for the CT scan, CT results, including incidental findings and systematic commentary on abdominal anatomy, and the format of the report were also recorded.

Data synthesis and statistical analyses

All categorical variables were summarised using absolute and relative frequencies. Continuous variables were summarised using the median (minimum-maximum). Logistic regression was used to compare categorical variables. Regression coefficients (B), standard errors (SE), odds ratios (OR), 95% confidence interval of the odds ratios (95% CI), and the p-values (p) were calculated. All statistical analyses were performed with a 95% confidence interval. Statistical significance was defined as a p-value of less than 0.05. The data analyses were conducted using IBM SPSS Statistics software, version 29.0.1.0 (IBM Corp., Armonk, NY).

## Results

Patient characteristics

A total of 192 abdominal CT scan reports identified between August 2023 and October 2023 met the inclusion criteria. Of these scans, 109 (56.8%) were performed on male patients. The median age at the time of the CT scan was 59 years (range: 18-97 years). Locally employed radiologists reported 113 scans (58.9%), while 79 scans (41.1%) were reported by outsourced radiology services. Incidental findings were noted in 36 scans (18.8%). The most common indication for CT was suspected appendicitis (30 (15.6%)), followed by abdominal pain (28 (14.6%)). Three scans (1.6%) did not have a documented indication. No abnormalities were detected in 42 scans (21.9%), and appendicitis was diagnosed in 21 scans (10.9%). The free-text reporting format was used in 149 scans (77.6%) (Table 1).

**Table 1:** Baseline characteristics of patients and abdominal CT scan reports The table summarises patient demographics, reporting formats, and the personnel responsible for interpreting the scans, as well as the indications for abdominal CT and the findings from the scans. Frequencies and percentages are provided for each category. Additionally, the table details the number and percentage of reports that included comments on key anatomical structures, such as the liver, biliary tree, spleen, pancreas, and others. It also indicates the number of incidental findings recorded. SD: standard deviation; CT: computed tomography; PR: per rectal

Patient and report characteristics	Total number = 192
Age (years)	
Mean ± SD	56.27 ± 17.67
Median (range)	59 (18-97)
Sex	
Female	109 (56.8%)
Male	83 (43.2%)
Reporting format	
Free-text	149 (77.6%)
Structured	43 (22.4%)
Reporting personnel	
Locally employed radiologists	113 (58.9%)
Outsourced reporting service	79 (41.1%)
Indication for CT scan	
Appendicitis	30 (15.6%)
Abdominal pain	28 (14.6%)
Bowel obstruction	27 (14.1%)
Gallbladder and biliary system pathology	18 (9.4%)
Abdominal wall hernias (complicated and uncomplicated)	16 (8.3%)
PR bleed	10 (5.2%)
Post-op collection	10 (5.2%)
Malignancy	9 (4.7%)
Diverticular disease (complicated and uncomplicated)	9 (4.7%)
No specified indication	3 (1.6%)
Others	32 (16.6%)
CT results	
No abnormality detected	42 (21.9%)
Acute appendicitis	21 (10.9%)
Gallbladder and biliary system pathology	18 (9.4%)
Diverticular disease (complicated and uncomplicated)	12 (6.3%)
Inconclusive results	15 (7.8%)
Abdominal wall hernias (complicated and uncomplicated)	16 (8.3%)
Bowel obstruction (small and large bowel)	16 (8.3%)
Pancreatitis	7 (3.6%)
Colitis	8 (4.2%)
Malignancy	5 (2.6%)
Others	32 (16.6%)
Anatomical structures commented on	
Liver	172 (89.6%)
Biliary system	173 (90.1%)
Spleen	167 (87.0%)
Pancreas	170 (88.5%)
Adrenals	166 (86.5%)
Kidneys	170 (88.5%)
Gastrointestinal tract	176 (91.7%)
Lymph nodes	147 (76.6%)
Mesentery and peritoneum	56 (29.2%)
Vasculature	87 (45.3%)
Bones and soft tissue	164 (85.4%)
Lung bases	168 (87.5%)
Incidental findings	36 (18.8%)

Comparison of abdominal CT scan reports: outsourced vs. locally employed radiologists

This study found that the outsourced radiology service was significantly less likely to report on several important intra-abdominal structures when compared to the locally employed radiologists. These structures included the biliary system (OR = 0.396, p = 0.046), spleen (OR = 0.414, p = 0.044), kidneys (OR = 0.354, p = 0.027), gastrointestinal tract (OR = 0.286, p = 0.026), lymph nodes (OR = 0.412, p = 0.011), mesentery and peritoneum (OR = 0.364, p = 0.004), vasculature (OR = 0.381, p = 0.002), bones and soft tissues (OR = 0.273, p = 0.003), as well as lung bases (OR = 0.300, p = 0.009).

While the outsourced service was less likely to comment on the pancreas (OR = 0.439, p = 0.075) and adrenals (OR = 0.460, p = 0.070), these differences were not statistically significant. However, there was no significant difference in reporting on the liver (OR = 0.839, p = 0.712) or other incidental findings (OR = 1.181, p = 0.656) (Table 2).

**Table 2:** Logistic regression analysis of abdominal CT reports: locally employed vs. outsourced radiologists This table shows the logistic regression analysis results comparing the likelihood of various abdominal structures being reported on by locally employed radiologists vs. outsourced radiology services. The table includes the number of scans with comments on each anatomical structure based on reporting personnel, logistic regression coefficients (B), standard errors (SE), odds ratios (OR), 95% confidence intervals (CI), and p-values. Statistically significant differences are indicated by p-values less than 0.05. N: number; B: regression coefficient; SE: standard error; OR: odds ratio; CI: confidence interval; GIT: gastrointestinal tract

Anatomical structures commented on	Locally employed (N=113)	Outsourced reporting (N=79)	B	SE	OR (95% CI)	p-value
Liver	102 (90.3%)	70 (88.6%)	-0.176	0.476	0.839 (0.330 - 2.130)	0.712
Biliary system	106 (93.8%)	67 (84.8%)	-0.998	0.501	0.369 (0.138 - 0.983)	0.046
Spleen	103 (91.2%)	64 (81%)	-0.881	0.438	0.414 (0.175 - 0.978)	0.044
Pancreas	104 (92%)	66 (83.5%)	-0.822	0.461	0.439 (0.178 - 1.085)	0.075
Adrenals	102 (90.3%)	64 (81%)	-0.776	0.428	0.460 (0.199 - 1.064)	0.070
Kidneys	105 (92.9%)	65 (82.3%)	-1.039	0.470	0.354 (0.141 - 0.890)	0.027
GIT	108 (95.6%)	68 (86.1%)	-1.251	0.561	0.286 (0.095 - 0.860)	0.026
Lymph nodes	94 (83.2%)	53 (67.1%)	-0.887	0.347	0.412 (0.209 - 0.814)	0.011
Mesentery and peritoneum	42 (37.2%)	14 (17.7%)	-1.010	0.353	0.364 (0.182 - 0.727)	0.004
Vasculature	62 (54.9%)	25 (31.6%)	-0.965	0.307	0.381 (0.209-0.695)	0.002
Bones and soft tissues	104 (92%)	60 (75.9%)	-1.297	0.436	0.273 (0.116 - 0.642)	0.003
Lung bases	105 (92.9%)	63 (79.7%)	-1.204	0.461	0.300 (0.121 - 0.741)	0.009
Incidental findings	20 (17.7%)	16 (20.3%)	0.166	0.373	1.181 (0.569 - 2.453)	0.656

Comparison of abdominal CT scan reports: free-text vs. structured formats

**Figure 1:**
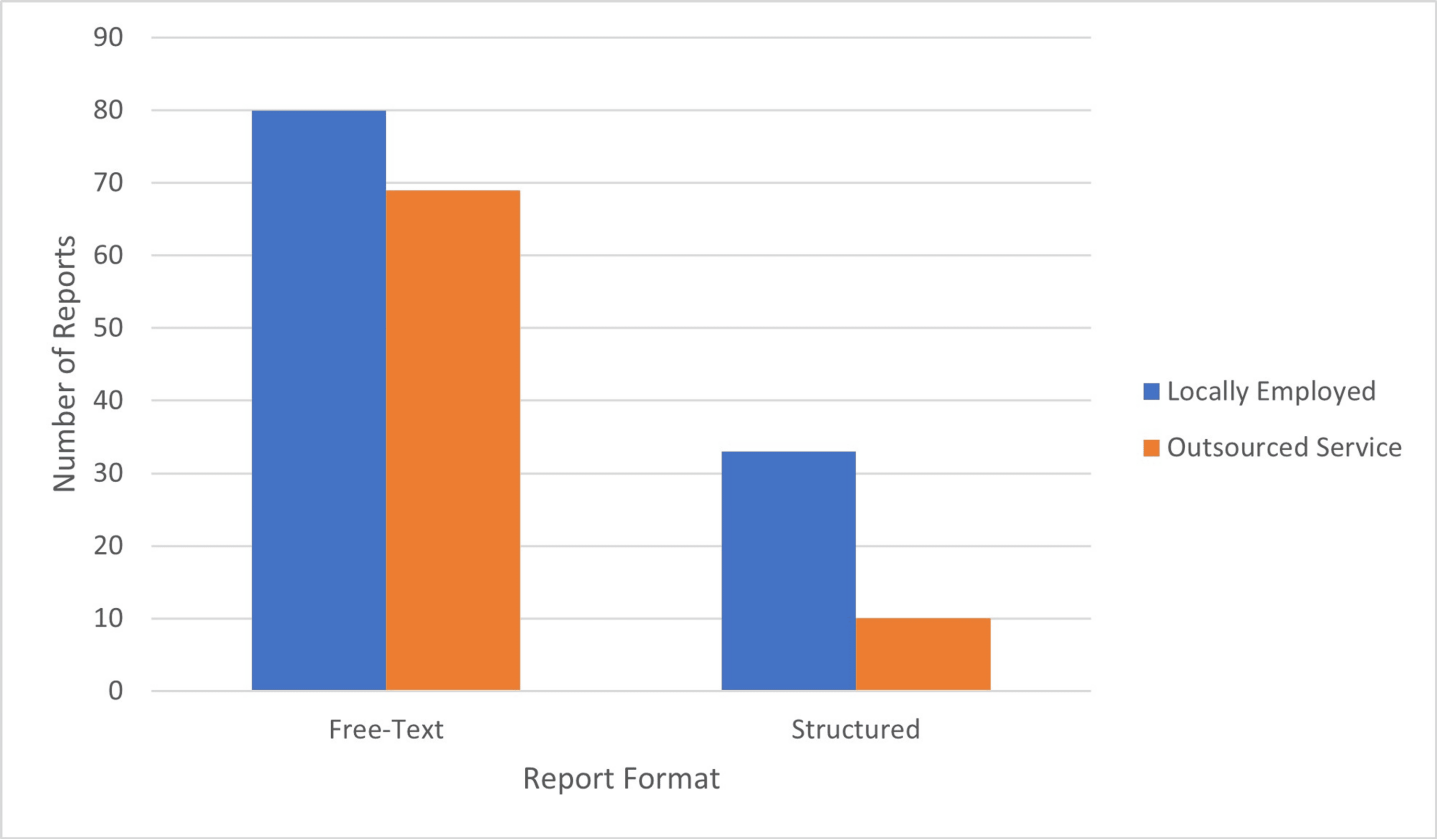
Comparison of reporting personnel by report format The bar chart illustrates the distribution of abdominal CT scan reports based on the personnel responsible for interpreting the reports (locally employed radiologists vs. outsourced radiology services) and the format used (free-text vs. structured), where blue bars represent locally employed radiologists and orange bars represent outsourced reporting services, with the y-axis showing the count of reports and the x-axis distinguishing between free-text and structured formats.

This study compared abdominal CT reports written in free-text and structured formats using logistic regression analysis. Of the structured reports, 33 (76.7%) were written by locally employed radiologists, while 10 (23.3%) were written by the outsourced radiology reporting service. Conversely, similar proportions of free-text reports were generated by locally employed radiologists (80 (53.7%)) and the outsourced service (69 (46.3%)) (Figure 1).

Structured reports were more likely to document most intra-abdominal structures, including incidental findings, compared to free-text reports. However, some of these findings were not statistically significant. The structured reports were significantly more likely to include comments on the following structures: spleen (OR = 8.064, p = 0.044), adrenals (OR = 8.468, p = 0.039), mesentery and peritoneum (OR = 1.949, p = 0.057) and bones and soft tissues (OR = 9.295, p = 0.031). While trends suggested that structured reports were more likely to include comments on the biliary system, pancreas, vasculature, and incidental findings, these differences were not statistically significant. Additionally, structured reports consistently included comments on the liver, kidneys, gastrointestinal tract, lymph nodes, and lung bases, leading to extremely high ORs without calculable confidence intervals (Table 3).

**Table 3:** Logistic regression analysis of abdominal CT report format: free-text vs. structured formats This table presents the results of a logistic regression analysis comparing abdominal CT scan reports written in free-text versus structured formats. It includes counts of reports with and without comments on key intra-abdominal structures for both formats. The analysis provides regression coefficients (B), standard errors (SE), odds ratios (OR), and 95% confidence intervals (95% CI) for each structure (some of which were incalculable), alongside p-values indicating statistical significance. Statistically significant values are indicated with p < 0.05. N: number; B: regression coefficient; SE: standard error; OR: odds ratio; CI: confidence interval; GIT: gastrointestinal tract

Anatomical structures commented on	Free-text (N = 149)	Structured (N = 43)	B	SE	OR (95% CI)	p-value
Liver	129 (86.6%)	43 (100%)	19.339	6129.370	250461215.946 (incalculable)	0.997
Biliary system	131 (87.9%)	42 (97.7%)	1.753	1.043	5.771 (0.748 - 44.536)	0.093
Spleen	125 (83.9%)	42 (97.7%)	2.087	1.036	8.064 (1.058 - 61.443)	0.044
Pancreas	128 (85.9%)	42 (97.7%)	1.930	1.039	6.891 (0.899 - 52.789)	0.063
Adrenals	124 (83.2%)	42 (97.7%)	2.136	1.035	8.468 (1.113 - 64.421)	0.039
Kidneys	127 (85.2%)	43 (100%)	19.450	6129.370	279846035.769 (incalculable)	0.997
GIT	133 (89.3%)	43 (100%)	19.085	6129.370	194342838.238 (incalculable)	0.998
Lymph nodes	104 (69.8%)	43 (100%)	20.365	6129.370	699003537.772 (incalculable)	0.997
Mesentery and peritoneum	32 (21.5%)	24 (55.8%)	1.530	0.366	4.618 (2.253 - 9.467)	0.000
Vasculature	62 (41.6%)	25 (58.1%)	0.667	0.351	1.949 (0.980 - 3.877)	0.057
Bones and soft tissues	122 (81.9%)	42 (97.7%)	2.229	1.034	9.295 (1.225 - 70.526)	0.031
Lung bases	125 (83.9%)	43 (100%)	19.553	6129.370	310171169.827 (incalculable)	0.997
Incidental findings	24 (16.1%)	12 (27.9%)	0.701	0.407	2.016 (0.909 - 4.472)	0.085

## Discussion

Outsourced radiology reporting has become a valid resource utilised worldwide [5]. It enables efficient reporting of time-critical scans performed in emergency care [5]. Previous studies have shown that outsourced reporting services handle a higher volume of scans compared to local radiology reporting services [10]. However, there is an increased risk of diagnostic errors, given the lack of direct communication between the outsourced radiologist and the clinical team [5].

In this study, emergency abdominal CT scan reports from the outsourced radiology reporting service were found to underreport critical information about various intra-abdominal organs. Specifically, outsourced reports frequently omitted comments on the biliary system, spleen, kidneys, lymph nodes, mesentery, peritoneum, vasculature, bones, soft tissues, and lung bases. As a result, this raises significant concerns regarding the possible omission of important scan findings. These findings align with those of Hohmann et al., who reported similar concerns in their prospective study at University College London Hospitals, where the rate of diagnostic errors in outsourced scan reports was 0.8% [11]. In 2023, Çetin et al. demonstrated comparable concerns regarding the diagnostic accuracy of outsourced thoracic CT scan reports in a Turkish cohort of patients [5].

In a 2018 survey, Graham et al. highlighted that local clinical teams frequently distrust the precision of outsourced CT scan reports [12]. More importantly, they highlighted that report clarifications and reviews were undertaken by locally employed radiologists when requested [12]. This contributes to a higher workload for local radiology teams, resulting in work duplication and inefficiencies in patient care [12]. Conversely, Olofsson et al. reported that fewer scans reported by locally employed radiologists needed reassessment compared to outsourced radiology services [13]. They recommended that clear communication with the outsourced radiology team is needed to guarantee a cost-effective service, shorter reporting time, and efficient management of patients [13]. In contrast, Storjohann et al. analysed 7761 out-of-hours CT scan reports, over 21 months, reported by locally employed and outsourced radiologists [14]. They concluded that there were no significant discrepancies in the accuracy of scan reports between the two groups [14].

This study also compared the effect of structured and free-text reporting formats on report comprehensiveness. According to the analyses, the free-text format was found to be more likely to underreport some of the important anatomical structures seen in abdominal CT scans. Notable examples of frequently omitted structures include the spleen, adrenal glands, mesentery and peritoneum, and bones and soft tissues. Several studies have compared structured and free-text reporting formats. Lam et al. described free-text CT reports as being more ambiguous compared to structured reports [15]. McFarland et al. and Dimarco et al. noted that the structured reporting style was superior and less prone to errors than the free-text style [7,16]. Errors in the free-text style may lead to misdiagnosis and unnecessary intervention [7,16]. Similarly, Jorg et al. acknowledged that structured reports are more concise [17]. However, they highlighted that the structured format is limiting when unexpected findings are uncovered [17]. Thus, a free-text format facilitates a more expansive description of uncertain findings [17].

The European Society of Radiology recommended the structured reporting format in Good Practice for Radiological Reporting in 2011 [18]. They highlighted that the structured format prompts radiologists to complete all required fields [18]. It is also suggested that the structured format is more time-efficient and enables easier retrieval of data for audit and research purposes [18].

This study has several limitations. Its retrospective design is a potential source of inherent biases, and being conducted at a single centre further increases the risk of these biases. Additionally, it was limited to abdominal CT scans, which may reduce the generalisability of findings to other imaging modalities. Furthermore, the CT images were not re-evaluated by independent radiologists, which could have helped identify potential omissions in the scan reports. Lastly, the relatively small sample size may also limit the strength of the conclusions drawn.

## Conclusions

In conclusion, this study highlights that the outsourced radiology service significantly underreported many key elements of abdominal CT scans when compared to the local radiology service. This underreporting can complicate the assessment and management of acute surgical patients. Additionally, the free-text reporting format was also found to omit critical details of abdominal CT scans. Further studies auditing a broader range of outsourced radiology services are thus recommended. This is essential to ensure that treating clinicians receive high-quality reports, enabling effective and efficient emergency surgical care.
